# The Impact of Childhood Emotional Abuse and Experiential Avoidance on Maladaptive Problem Solving and Intimate Partner Violence

**DOI:** 10.3390/bs5020154

**Published:** 2015-04-16

**Authors:** Kathryn M. Bell, Lorrin Higgins

**Affiliations:** Department of Psychology, Capital University, 1 College & Main, Columbus, OH 43209, USA; E-Mail: lhiggins@capital.edu

**Keywords:** intimate partner violence, childhood emotional abuse, experiential avoidance, social problem solving

## Abstract

The purpose of the current study was to examine the joint influences of experiential avoidance and social problem solving on the link between childhood emotional abuse (CEA) and intimate partner violence (IPV). Experiential avoidance following CEA may interfere with a person’s ability to effectively problem solve in social situations, increasing risk for conflict and interpersonal violence. As part of a larger study, 232 women recruited from the community completed measures assessing childhood emotional, physical, and sexual abuse, experiential avoidance, maladaptive social problem solving, and IPV perpetration and victimization. Final trimmed models indicated that CEA was indirectly associated with IPV victimization and perpetration via experiential avoidance and Negative Problem Orientation (NPO) and Impulsivity/Carelessness Style (ICS) social problem solving strategies. Though CEA was related to an Avoidance Style (AS) social problem solving strategy, this strategy was not significantly associated with IPV victimization or perpetration. Experiential avoidance had both a direct and indirect effect, via NPO and ICS social problem solving, on IPV victimization and perpetration. Findings suggest that CEA may lead some women to avoid unwanted internal experiences, which may adversely impact their ability to effectively problem solve in social situations and increase IPV risk.

## 1. Introduction

Childhood maltreatment is a significant problem within the United States. One nationally representative sample found that one in eight children reported some form of childhood maltreatment during the study year, with childhood emotional abuse (CEA) being most commonly experienced [1]. Another study examining prevalence rates of childhood maltreatment in a large community sample found that about 17% of women and 21% of men reported childhood physical abuse (CPA), nearly 8% of women and approximately 2% of men experienced childhood sexual abuse (CSA), and approximately 14% of women and nearly 10% of men reported experiencing CEA. In approximately 13% of cases, multiple forms of childhood maltreatment were reported [2].

Although CEA is one of the most commonly reported forms of childhood maltreatment, it is understudied and less understood than other forms of childhood abuse [3,4,5]. Difficulties operationally defining CEA and perceived lack of intent to harm a child, despite harm occurring, may contribute to the under-recognition of CEA in the child maltreatment field [4]. CEA is sometimes referred to in relation to similar constructs such as “psychological maltreatment”, “emotional maltreatment”, and “psychological abuse”. Psychological maltreatment, broadly defined, is characterized by parental behaviors that ignore or harm a child’s psychological or emotional needs, including acts of ignoring, terrorizing, exploiting, corrupting, neglecting, or rejecting a child [3,4]. CEA, more narrowly defined for the purposes of the current study, is defined as “verbal assaults on a child’s sense of worth or well-being, or any humiliating, demeaning, or threatening behavior directed toward a child by an older person” [6]. U.S. child welfare statistics indicate rates of psychological maltreatment ranging from 7.8%–9.0% between 2009 and 2013 [7]. These national statistics likely underestimate rates of psychological maltreatment due to difficulties defining and recognizing psychological maltreatment, especially in situations where other forms of childhood abuse or neglect are present [4]. A meta-analysis of 29 studies estimating worldwide CEA prevalence rates found a combined CEA worldwide prevalence of 26.7%, with higher prevalence rates found in self-report studies in comparison to informant-report studies [8]. The World Health Organization notes the difficulties, however, in global definitions of CEA, as childrearing and disciplinary practices may vary across cultures and differentially impact children. For example, threats of abandonment are more commonly reported as a disciplinary strategy used by mothers in the Philippines in comparison to Chilean mothers [9]. As such, some have argued that CEA definitions need to consider both the act and impact on the child [10]. Definitional, measurement, and cross-cultural variations make it difficult to determine the most common types of CEA. An international survey indicated that yelling is a common verbal or psychological punishment reported by mothers across numerous countries [9]. Another study conducted in the United States with children recruited from a child welfare agency found that terrorizing, including threats against the child or child’s loved ones, domestic violence exposure, and placement in unpredictable or chaotic situations that are recognizably dangerous, was the most commonly identified form of CEA [11]. CEA can occur in isolation but often transpires in conjunction with other forms of childhood maltreatment [12,13,14]. When models include the effects of multiple forms of childhood maltreatment, including CEA, CSA, and CPA, CEA often remains a strong predictor of short and long-term impairments [5,12,15].

CEA is associated with numerous adverse outcomes in adulthood including depression, anxiety, dissociation, somatization, low self-esteem, anger/irritability, and marital dissatisfaction [16,17,18,19]. Harter suggested that childhood maltreatment, especially CEA, can impair children’s self-system, including self-awareness, agency, self-coherence, and self-continuity, which can adversely impact children’s self-esteem and enhance feelings of guilt, shame, depression, and anger [20]. Diagnoses of posttraumatic stress disorder (PTSD), eating disorders, and substance use disorders are commonly reported by individuals with a CEA history [21,22,23]. CEA can also lead to long-term impairments in interpersonal functioning [3]. Interpersonal difficulties associated with CEA include social isolation, codependency, poor relationship quality, loneliness, social distancing, conflict avoidance, and increased interpersonal conflict [3,24,25,26,27]. CEA may also increase risk for intimate partner violence (IPV) within adolescent and adult relationships.

### 1.1. Childhood Emotional Abuse and Physical IPV

A growing body of evidence suggests that individuals who experience childhood maltreatment are at an increased risk for IPV perpetration and victimization in adolescence and adulthood [28,29]. Although understudied relative to some other forms of childhood maltreatment, preliminary evidence suggests that CEA may significantly impact future IPV risk [3,15]. For example, a study of physically abused women, maritally discordant, non-abused women, and maritally satisfied women found that physically abused women and martially discordant women reported higher CEA rates than maritally satisfied women, and physically abused women reported the highest CEA rate in comparison to the other two groups [30]. A similar predictive relationship between CEA and IPV victimization was found in a representative community sample of women, although CSA and low educational attainment were also predictive of IPV victimization in this sample [31]. Wekerle and colleagues [32] found that CEA was predictive of IPV perpetration and victimization among adolescent males but was only predictive of IPV victimization among adolescent females. Additional studies with college-aged men and women have also found CEA to be predictive of both IPV victimization and perpetration [33,34]. Notably, in one study with college-aged men and women, CEA was predictive of IPV victimization and perpetration even after controlling for other forms of childhood maltreatment and accounted for more variance in IPV victimization than CPA [34].

Although preliminary evidence suggests a predictive relationship between CEA and IPV, the mechanisms underlying this relationship are not well-understood. Berzenski and Yates [15] suggest that poor coping skills that develop following CEA exposure may account for the relationship between CEA and IPV. Specifically, the authors propose that CEA interferes with the childhood development of adaptive coping strategies, such as emotion regulation skills. These skills may be especially compromised in heightened emotional contexts. Additionally, individuals exposed to CEA may experience impairments in social cognition that interfere with interpersonal functioning. The combination of poor emotional regulatory coping skills and impaired social cognition in emotionally charged interpersonal situations may increase risk for negative social consequences, including IPV [15].

Conceptual models of information-processing systems may provide some theoretical support for the roles of emotional regulatory coping skills and social cognition in explaining the link between CEA and IPV. As summarized by D’Zurilla and Maydeu-Olivares [35], two different information-processing systems may operate in coping with stressful situations. The automatic or experiential system is believed to drive much of daily coping behavior and is characterized as emotional, unintentional, and intuitively driven that often results in immediate action. Alternatively, the nonautomatic/rational system is slower, logical, analytic, and deliberate, resulting in intentional action determined through the use of problem solving techniques and may override more automatic processes. Consideration of both emotional and cognitive processes in coping with stressful situations may be necessary to better understand negative consequences resulting from ineffective coping.

Lemerise and Arsenio [36] maintained that both emotion and cognitive processes impacting social development in childhood should be considered. They argue that emotion processes serve motivational, regulatory, and communicative functions that are distinct from the cognitive processes involved in social competence. These emotion processes may impact the cognitive processes of decision-making and goal selection. For example, children with poor emotional regulatory skills may choose avoidant or hostile goals when faced with an emotionally overwhelming social situation in order to temporarily reduce aversive arousal [36]. Poor emotional regulatory skills may interfere with children’s ability to flexibly approach decision-making and goal selection by considering multiple perspectives and contextual factors [37]. Taken together, the authors argue that systematic attempts be taken to integrate affect and cognition in future models of social competence, socio-moral development, and psychopathology.

Given that both emotion and cognitive processes may be affected by childhood stressors and impact later social functioning, these two processes should be considered when investigating possible mechanisms underlying the CEA-IPV link. It is possible that affective and cognitive impairments that arise following CEA could jointly contribute to IPV risk under emotionally-provocative interpersonal situations. Thus, the current study chose to examine the role of both emotional and social cognitive factors, experiential avoidance and social problem solving, on the relationship between CEA and IPV.

### 1.2. Experiential Avoidance as a Mechanism of CEA-IPV Link

Experiential avoidance is a process in which a person is unwilling to experience and attempts to avoid unwanted private experiences (e.g., distressing thoughts, sensations, memories or emotions) [38]. Experiential avoidance is associated with psychological distress and numerous clinical problems including substance abuse, somatization, self-harm behaviors, depression, anxiety, and PTSD [39,40,41]. A limited number of studies have also identified a relationship between experiential avoidance and aggression, including interpersonal violence. A study of male students, faculty, and staff found that experiential avoidance was predictive of aggressive behavior even after controlling for the effects of trait anger and PTSD symptom severity [42]. Another study of 49 male soldiers and their female partners found that experiential avoidance was associated with an increase in physical IPV perpetration and victimization among men [43]. No known studies, however, have examined the impact of experiential avoidance on IPV perpetration and victimization within community and female samples.

Although research on the relationship between childhood maltreatment and experiential avoidance is also limited, preliminary findings indicate that individuals who experience childhood maltreatment may exhibit heightened levels of experiential avoidance and emotional inhibition [44]. A study of 100 adolescent females, some with a childhood maltreatment history, found heightened experiential avoidance among those reporting childhood maltreatment [45]. Findings from a community sample of 127 individuals ages 18–30 indicated that exposure to emotional invalidation in childhood, a characteristic of CEA, was associated with chronic emotional inhibition in adulthood. Chronic emotional inhibition was characterized by thought suppression, avoidant stress responses, and ambivalence over emotional expression [46]. Lastly, a study by Gratz and colleagues [44] examined the relationships between different forms of childhood abuse, including CPA, CSA, and CEA, experiential avoidance, and emotional nonacceptance among a sample of inner-city substance users. The authors discovered heightened experiential avoidance among individuals reporting moderate to severe CPA, CSA, and CEA. Notably, only CSA and CEA accounted for unique variance in emotional nonacceptance above and beyond that accounted for by current major depression. Additionally, emotional nonacceptance only mediated the relationship between CEA and experiential avoidance. Taken together, these initial findings suggest that childhood maltreatment, especially CEA, can heighten emotional nonacceptance, emotional inhibition, and experiential avoidance. Additional research is needed to further investigate the relationships between different forms of childhood abuse and experiential avoidance [44].

No known studies have examined the extent to which experiential avoidance mediates the relationship between CEA and IPV. Furthermore, only one known study has investigated emotion dysregulation, a construct related to experiential avoidance, as a mediator of the relationship between childhood maltreatment and relational aggression. Gratz and colleagues [47] investigated emotion dysregulation as a possible mediator of the relationship between childhood maltreatment and physical IPV perpetration among male and female college students. The authors found that emotion dysregulation mediated the relationship between childhood maltreatment and physical IPV perpetration among men. This mediational relationship was not found for women. The study, however, was limited in scope. Different forms of childhood maltreatment, including CEA, and their impact on emotion dysregulation and subsequent IPV perpetration were not examined. Additionally, the study did not investigate emotion dysregulation as a mediator of the relationship between childhood maltreatment and IPV victimization. As such, additional research is needed to examine how different forms of childhood maltreatment impact emotion processes, including experiential avoidance, and subsequent IPV perpetration and victimization. Furthermore, it is possible that experiential avoidance that develops from CEA could indirectly impact IPV perpetration and victimization through additional pathways not yet examined. Future research is needed to examine how other coping processes, such as social-problem solving skills, are affected by experiential avoidance stemming from CEA and can lead to IPV perpetration and victimization.

### 1.3. Social Problem Solving as a Mechanism of CEA-IPV Link

Social problem solving is a self-directed cognitive behavioral process in which a person attempts to identify adaptive or effective strategies for coping with everyday problems in living [35,48]. Social problem solving is considered to be purposeful, rational, and effortful [35]. Findings from factor-analytic studies indicate that social problem solving is a multi-dimensional construct consisting of five different problem solving dimensions [49,50]. The five dimensions include Positive Problem Orientation (PPO; cognitive set in which problems viewed as a solvable challenge that can be solved effectively using one’s own abilities), Negative Problem Orientation (NPO; ineffective or dysfunctional cognitive-emotional set in which problems are viewed as threatening and unlikely to be solved using one’s personal abilities, leading to frustration and distress when encountering problems), Rational Problem Solving (RPS; constructive problem solving style characterized by rational, systematic, and deliberate use of problem solving skills), Impulsivity/Carelessness Style (ICS; maladaptive problem solving style in which problem solving skills are applied carelessly, impulsively, narrowly, and incompletely), and Avoidance Style (AS; dysfunctional problem solving style characterized by passivity, dependency, inaction, and procrastination). Individuals are considered to have “good” social problem solving ability if they demonstrate high PPO and RPS and low ICS, AS, and NPO. Poor social problem solving ability is thought to be reflected by high ICS, AS, and NPO and low PPO and RPS [51]. Social problem solving deficits are associated with heightened psychological distress, including depression and anxiety, and effective social problem solving ability has been shown to weaken the impact of stressful life events on psychological adjustment [52,53,54,55,56,57]. Effective social problem solving has also been found to be positively associated with positive psychological well-being [48,58]. Lastly, relational dysfunction and marital distress have been shown to be associated with deficits in interpersonal problem solving [59,60].

Life stress appears to reciprocally influence the occurrence of daily problems, psychological distress, and problem solving skills [61]. Major stressful life events (e.g., injury or death of loved one, serious medical condition) can increase the occurrence of daily problems, which can subsequently trigger additional major life stressors and daily problems. Psychological distress may arise in reaction to major stressful life events, daily problems, and ineffective attempts to problem solve. Severe psychological distress may also impair one’s ability to effectively cope and problem solve, making it difficult to effectively solve daily problems that arise and potentially increase the severity and frequency of daily problems, which may heighten risk for additional major life stressors [61]. It is possible that CEA, and related stressors (e.g., involvement in child welfare services, disruptive home environments), may increase the presence of daily problems during childhood. Psychological distress experienced in reaction to these stressors and daily problems may interfere with children’s abilities to effectively cope and problem solve, which may further heighten their psychological distress and increase exposure to additional problems and major life stressors. Continued exposure to major life stressors, daily problems, and severe psychological distress during childhood and adolescence may further impair problem solving skills, which may lead to ineffective problem solving and greater risk for daily problems and major life stressors in adulthood.

Aggressive and violent behavior may be a maladaptive attempt to solve an interpersonal problem [62]. Studies with children and adolescents have found a significant association between social problem solving deficits and aggressive behavior [63,64,65]. Few studies, however, have examined the impact of social problem solving deficits on relational conflict and aggressive behavior among adults. In a study of 205 college students, poor constructive problem solving (as reflected by low PPO and RPS scores) and heightened maladaptive problem solving (as indicated by high NPO, AS, and ICS scores) were found to be associated with more hostility [62]. Greater maladaptive problem solving was also related to increased anger, but only an impulsive/carelessness problem solving style (as reflected by a high ICS score) was significantly predictor of aggressive behavior as measured by the Aggression Questionnaire [62,66]. Another study conducted with 123 college students found that constructive interpersonal problem solving (*i.e.*, problem solving of interpersonal conflict) was negatively associated with interpersonal conflict and maladaptive interpersonal problem solving was positively correlated with interpersonal conflict [67]. Taken together, results from the two studies suggest that maladaptive problem solving, especially an impulsive/carelessness problem solving style, may increase risk for aggressive behavior and relational conflict. Neither study, however, assessed the impact of social problem solving on risk for IPV perpetration and victimization. As such, additional research is needed to investigate the extent to which maladaptive social problem solving predicts IPV perpetration and victimization. Additionally, research is needed to examine the potential mediating role of social problem solving in the relationship between emotion processes, such as experiential avoidance, and aggression [62].

### 1.4. Summary and Current Study

Although CEA is a commonly reported form of childhood maltreatment that often co-occurs with other forms of childhood maltreatment, it is not well-researched and little is known about the long-term effects of CEA on interpersonal functioning after controlling for the effects of other forms of childhood maltreatment [5,26]. Numerous researchers have called for studies that investigate the unique impact of CEA on long-term outcomes after controlling for the effects of other types of childhood maltreatment [5,15,26,44,68]. Preliminary studies suggest that CEA may predict IPV perpetration and victimization in adulthood [3,30,31,32,33,34]. The mechanisms underlying the relationship between CEA and IPV, however, are not well-researched or understood [3,26,33]. It is possible that both affective and cognitive coping processes, such as experiential avoidance and social problem solving, jointly contribute to the relationship between CEA and IPV perpetration and victimization [62,69]. Yet, little is known about the role of experiential avoidance in the relationship between CEA and IPV. Furthermore, no known studies have investigated the extent to which experiential avoidance stemming from CEA impacts social problem solving and subsequent IPV perpetration and victimization.

Therefore, the purpose of the current study was to: (a) examine the unique impact of CEA, after controlling for the effects of CSA and CPA, on IPV perpetration and victimization among a female community sample; and (b) use path analytic modeling to investigate the joint influences of experiential avoidance and maladaptive social problem solving on the relationship between CEA and IPV perpetration and victimization while controlling for the influences of CPA and CSA. It is hoped that results from the current study will shed additional light on the possible mechanisms accounting for the relationship between CEA and IPV.

The study hypotheses were as follows:

1. After including the influences of experiential avoidance and social problem solving, none of the childhood maltreatment forms would be directly associated with IPV victimization and perpetration.
2. Experiential avoidance would be indirectly associated with IPV victimization and perpetration via all three maladaptive social problem solving dimensions.
3. After controlling for the effects of CPA and CSA, CEA would remain indirectly predictive of IPV victimization and perpetration via experiential avoidance and social problem solving.

## 2. Method

### 2.1. Participants

Participants were 232 women recruited from a Midwestern community. Recruitment was done through flyer advertising posted throughout the community and through the local domestic assault adult shelter. Participants were selected based on their varied IPV victimization history, with 44.4% of the sample reporting an IPV victimization history in the past six months and 55.6% not reporting a history of IPV victimization in the past six months. Participants disclosed a range of IPV victimization and perpetration experiences occurring during the past year. The mean age of the sample was 31.96 (*SD* = 10.09). Participants identified as the following: 48.3% Caucasian, 37.9% African-American, 5.2% Biracial, 3.4% Latino/Latina, 1.7% Asian-American, 1.3% Native American, 1.7% “Other” and 0.4% unidentified. Of the 231 participants who reported on their current relationship status, 39.8% were single, 20.3% were living with an intimate partner, 26.8% were married, 2.2% were widowed, 5.6% were divorced, 0.4% were remarried, and 4.8% were separated. All of the female participants who reported IPV victimization in the past six months were with male partners at the time of the IPV episode. Nearly 42% of the sample reported attending some college or vocational training but did not complete a college degree. The majority of the sample (59.9%) reported not being employed at the time of this study. Nearly a quarter of participants (23.7%) indicated that they had ever gone to a “safe house” or battered women’s shelter.

### 2.2. Measures

#### 2.2.1. Demographic Questionnaire

Participants responded to a demographics questionnaire that assessed age, relationship status, educational history, employment status, and race/ethnicity.

#### 2.2.2. Physical Intimate Partner Violence

The Revised Conflict Tactics Scales (CTS2) measures the annual prevalence, chronicity and severity of different tactics used in response to conflict in an intimate relationship [70]. Participants respond to 78 items on an 8-point scale assessing their responses and their partners’ responses to relationship conflict during the past year across five domains: Negotiation, Psychological Aggression, Physical Assault, Sexual Coercion, and Injury. For the purposes of the current study, only the Physical Assault subscale was used to assess physical IPV perpetration and victimization. The Physical Assault subscale contains 12 items ranging from minor (e.g., “I pushed or shoved my partner”) to severe (e.g., “I choked my partner”) acts of physical aggression. Item response composite scores for physical assault were calculated separately for perpetration and victimization using the method described by Regan, Bartholomew, Kwong, Trinke, and Henderson [71]. Adequate internal consistencies for female reports of perpetration (α = 0.75) and victimization (α = 0.89) have been demonstrated using this scoring method [71].

#### 2.2.3. Social Problem Solving

The Social Problem-Solving Inventory-Revised Short Form (SPSI-R:S) [48] is a 25-item, multidimensional, self-report measure assessing individuals’ strengths and weaknesses in their social problem solving. Participants respond to each item on a 5-point Likert scale ranging from “0—Not at all true of me” to “4—Extremely true of me.” The measure is comprised of five different social problem solving dimensions consisting of Positive Problem Orientation (PPO), Negative Problem Orientation (NPO), Rational Problem Solving (RPS), Impulsivity/Carelessness Style (ICS), and the Avoidance Style (AS). Individuals are considered to have “good” social problem solving ability if they score high on PPO and RPS and low on ICS, AS, and NPO. Poor social problem solving ability is thought to be reflected by high scores on ICS, AS, and NPO and low scores on PPO and RPS [51]. Internal consistencies across the five scales range from 0.69 to 0.95, with the NPO, ICS, and RPS scales demonstrating the highest internal consistencies. Test-retest reliability coefficients have ranged from 0.68 to 0.91. The measure has also demonstrated adequate convergent validity, with all of SPSI-R:S scales correlating in the predicted directions with self-report measures of distress [48]. The measure has been shown to be distinguishable from measures assessing experiential coping [35]. The NPO, ICS, and AS scales were used in the current study.

#### 2.2.4. Childhood Abuse

The Childhood Trauma Questionnaire (CTQ) [6,72] is a 28-item self-report measure assessing retrospectively various forms of childhood abuse and neglect using a Likert-type scale ranging from “never true” to “very often true”. The CTQ consists of five subscales corresponding to childhood physical abuse, emotional abuse, sexual abuse, emotional neglect, and physical neglect. The CTQ physical abuse, emotional abuse, and sexual abuse subscales were used in the current study. Cronbach’s alphas range from 0.58 to 0.94 for each of the five factors, with the physical neglect subscale demonstrating the lowest internal inconsistency [2]. The measure has demonstrated strong test-retest reliability over an average 4-month period, with test-retest reliability coefficients ranging from 0.79 to 0.86 [6]. The measure has been shown to be strongly correlated with the Childhood Trauma Interview, suggesting that the CTQ has good convergent validity. Good discriminant validity has been demonstrated based on the CTQ’s comparisons with measures of verbal intelligence and social desirability scores [72].

#### 2.2.5. Experiential Avoidance

The Acceptance and Action Questionnaire (AAQ) is a general measure of experiential avoidance that assesses negative evaluations of internal experience, psychological acceptance or nonacceptance, and the extent to which a person acts when experiencing emotional distress [73]. The AAQ consists of 9-items that participants respond to using a 7-point scale ranging from “never true” to “always true”. The measure has demonstrated adequate test-retest reliability (0.64) and internal consistency (α = 0.70). The AAQ has been shown to be significantly correlated with measures of thought suppression and control, depression, psychological trauma, and anxiety [73].

### 2.3. Procedure

As part of a larger study on trauma and mental health, participants completed a series of questionnaires assessing demographic background, experiential avoidance, childhood abuse history, social problem solving, and IPV perpetration and victimization. Participants were recruited from the community by posting flyers in local community businesses (e.g., laundromats, grocery stores) and public/social service agencies (e.g., domestic assault shelter, police department, public libraries), placing advertisements in local newspapers, and contacting individuals who had previously participated in family violence research through our center and had agreed to be contacted for future research. The flyers and advertisements indicated that the study’s researchers were seeking participants over the age of 18 to participate in a study examining conflict in relationships and how adverse life experiences impact women’s mental health. Participants completed one in-person session, during which time they responded to questionnaires and participated in an interview that was administered as part of a larger study. Transportation and childcare assistance arrangements were made for participants as needed. Questionnaires were administered individually by trained female research assistants to reduce the likelihood of underreporting sensitive behaviors [74]. Participants were paid $50 for their involvement in the study and provided with a debriefing form at the end of their involvement in the study that included information on local social service resources. All study procedures were reviewed and approved by the IRB prior to the onset of data collection.

Descriptive statistics were computed for all demographic, independent, and dependent study variables. Correlation analyses were also performed on all of the independent and dependent study variables. A series of path analytic models were then conducted using the AMOS program [75]. A fully saturated model was estimated and then non-significant pathways were trimmed sequentially. Pathways were removed if the critical ratio (CR) was less than 1.65. Missing data was assumed to be missing at random and included in all estimates. Separate path analyses were performed for physical IPV perpetration and victimization.

## 3. Results

### 3.1. Descriptive Characteristics of Study Variables

Table 1 summarizes the study variable means, standard deviations, score ranges, and zero-order correlations. Mean number of physical IPV perpetration and victimization items endorsed was 20.05 (*SD* = 2.46) and 3.31 (*SD* = 3.70), respectively. Approximately 59% of the sample reported minor physical IPV perpetration and about 34% reported engaging in severe physical IPV perpetration in the past year. Minor physical IPV victimization was experienced by approximately 58% of the sample and about 47% of the sample reported experiencing severe physical IPV victimization in the past year. As expected, with the exception of childhood sexual abuse history and the Negative Problem Orientation social problem solving domain, physical IPV perpetration was positively correlated with childhood physical and emotional abuse, experiential avoidance, and Avoidance Style and Impulsivity/Carelessness social problem solving domains. Similarly, with the exception of the Negative Problem Orientation social problem solving domain, physical IPV victimization was positively correlated as anticipated with childhood physical, sexual, and emotional abuse, experiential avoidance, and Avoidance Style and Impulsivity/Carelessness social problem solving domains.

**Table 1:** Descriptive statistics and correlation matrix of study variables.

Variables	1	2	3	4	5	6	7	8	9
**N**	221	225	231	230	232	169	230	230	230
**M/SD**	2.05 (2.46)	3.31 (3.70)	10.81 (6.16)	9.23 (5.33)	8.75 (6.22)	35.11 (7.07)	98.17 (16.62)	97.89 (17.65)	97.67 (14.19)
**Min/Max**	0/12	0/12	5/25	5/25	5/25	16/54	73/162	74/162	78/155
1. Physical IPV Perpetration	1								
2. Physical IPV Victimization	0.66 **	1							
3. Childhood Emotional Abuse	0.16 *	0.21 **	1						
4. Childhood Physical Abuse	0.20 **	0.24 **	0.66 **	1					
5. Childhood Sexual Abuse	0.12	0.18 **	0.46 **	0.47 **	1				
6. Experiential Avoidance	0.24 **	0.28 **	0.20 **	0.14	0.04	1			
7. SPSI-R:S—Impulsivity/Carelessness (IC)	0.29 **	0.33 **	0.23 **	0.08	0.07	0.31 **	1		
8. SPSI-R:S—Negative Problem Orientation (NPO)	0.03	0.03	0.25 **	0.13	0.07	0.42 **	0.59 **	1	
9. SPSI-R:S—Avoidance Style (AS)	0.17 *	0.16 *	0.21 **	0.07	0.09	0.34 **	0.65 **	0.66 **	1

IPV = Intimate Partner Violence; SPSI-R: S = Social Problem-Solving Inventory-Revised: Short Form * < 0.05; ** < 0.01.

### 3.2. Path Analyses

**Figure 1:**
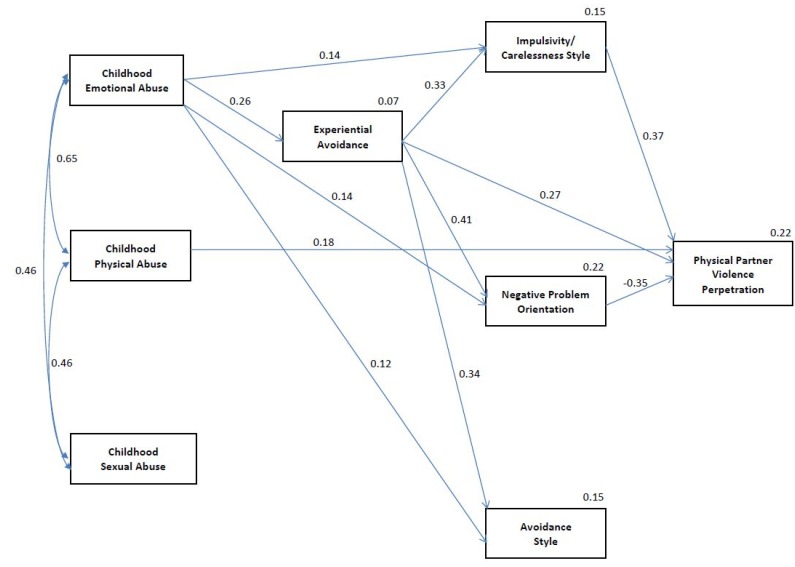
Final trimmed path analytic model of direct and indirect relationships between childhood abuse, experiential avoidance, social problem solving, and physical IPV perpetration.

Figure 1 presents the final trimmed model of childhood emotional, sexual, and physical abuse, experiential avoidance, and SPSI NPO, AS, and ICS domains predicting physical IPV perpetration. Following examination of the fully saturated model and the CRs related to each path, the paths from childhood sexual and physical abuse to experiential avoidance (β = −0.03, CR = −0.40 and β = −0.01, CR = −0.10, respectively), childhood sexual abuse to NPO, AS, and ICS (β = −0.04, CR = −0.50, β = 0.03, CR = 0.37, and β = −0.01, CR = −0.13, respectively), childhood physical abuse to NPO (β = −0.04, CR = −0.48), childhood emotional and sexual abuse to physical IPV perpetration (β = −0.04, CR = −0.47 and β = 0.03, CR = 0.37, respectively), and AS to physical IPV perpetration (β = −0.09, CR = 0.96) were removed. The remaining paths that were retained within the model all had CRs above 1.80. The final model had a non-significant chi-square statistic, χ^2^ = 4.72, *p* = 0.94. The final trimmed model indicated that only childhood physical abuse was directly and related to physical IPV perpetration. Childhood emotional abuse was indirectly associated with physical IPV perpetration via experiential avoidance, ICS, and NPO. Childhood emotional abuse and experiential avoidance were also associated with AS but AS was not significantly related to physical IPV perpetration. Childhood sexual abuse was not directly or indirectly associated with physical IPV perpetration. Experiential avoidance was directly and indirectly associated with physical IPV perpetration via both ICS and NPO.

**Figure 2:**
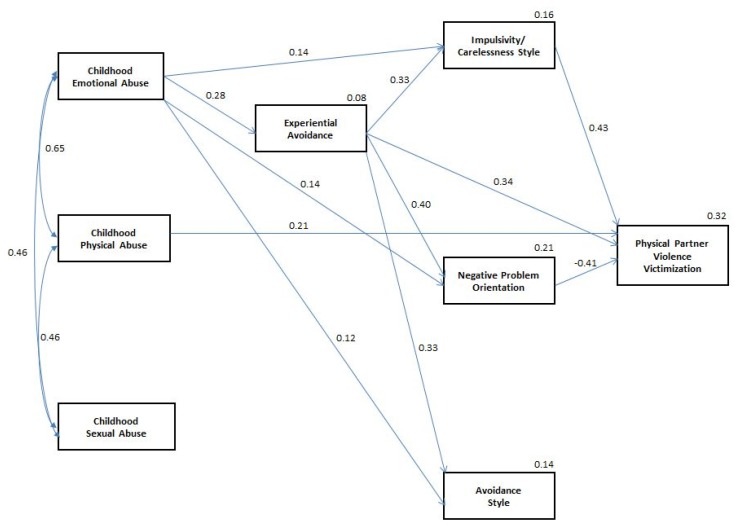
Final trimmed path analytic model of direct and indirect relationships between childhood abuse, experiential avoidance, social problem solving, and physical IPV victimization.

Similar path analyses were conducted for physical IPV victimization. Figure 2 presents the final trimmed model of childhood physical, emotional, and sexual abuse, experiential avoidance, and NPO, ICS, and AS social problem solving domains predicting physical IPV victimization. After examining the fully saturated model and the CRs associated with each path, the paths from childhood sexual and physical abuse to experiential avoidance (β = −0.04, CR = −0.45 and β = 0.00, CR = 0.02, respectively), childhood sexual abuse to NPO, AS, and ICS (β = −0.03, CR = −0.49, β = 0.03, CR = 0.38, and β = −0.01, CR = −0.10, respectively), childhood physical abuse to NPO (β = −0.04, CR = −0.52), childhood emotional and sexual abuse to physical IPV perpetration (β = −0.04, CR = −0.53 and β = 0.06, CR = 0.94, respectively), and AS to physical IPV perpetration (β = −0.01, CR = 0.09) were removed. The remaining paths that were retained within the model all had CRs above 1.80. The final model had a non-significant chi-square statistic, χ^2^ = 4.56, p = 0.95. Only childhood physical abuse and experiential avoidance were directly related to physical IPV victimization. Childhood emotional abuse was indirectly associated with physical IPV victimization via experiential avoidance, ICS, and NPO. Childhood sexual abuse was not directly or indirectly related to physical IPV victimization. Similar to the final trimmed model of physical IPV perpetration, childhood emotional abuse and experiential avoidance were associated with AS but AS was not significantly associated with physical IPV victimization.

## 4. Discussion

The purpose of the current study was to examine the extent to which experiential avoidance and maladaptive problem solving jointly contribute to the relationship between CEA and IPV victimization and perpetration after controlling for the effects of other forms of childhood maltreatment. The study was unique in investigating potential affective and cognitive mechanisms of action impacting the relationship between CEA and IPV. Results indicate that the study’s hypotheses were partially supported.

Zero-order correlations demonstrated significant positive correlations between all childhood maltreatment forms and IPV perpetration and victimization, with the exception of a non-significant relationship between CSA and IPV perpetration. When all forms of childhood maltreatment were included in the final models, however, CSA and CEA were no longer directly associated with IPV perpetration and victimization. CPA continued to have a direct effect, but no indirect effect via experiential avoidance or social problem solving, on IPV perpetration and victimization. Findings suggest that different mechanisms might play a role in explaining the relationship between CPA and IPV.

Interestingly, final models indicated that CSA was not directly or indirectly associated with IPV perpetration or victimization. This finding is inconsistent with some previous research indicating a positive association between CSA and IPV [76,77]. Several possible explanations might account for this discrepant finding. Although participants reported a range of CSA experiences, the mean CSA score was lower than the mean scores for CPA and CEA. Thus, it is possible that limited reporting of CSA experiences in the current study prevented detection of the direct and indirect impact of CSA on IPV. Alternatively, it could be that CPA and CEA experiences, which could co-occur with CSA experiences, have a greater long-term impact on interpersonal functioning. Lastly, the current study’s findings may reflect a gender-specific non-significant relationship between CSA and IPV perpetration and victimization. This explanation is supported by two recent studies that found that, after controlling for the effects of CPA and childhood neglect, CSA directly predicted male IPV perpetration but not female IPV perpetration [78,79]. Future research is needed to further investigate the differential effects of gender on the relationship between different forms of childhood maltreatment and IPV.

The second hypothesis, which predicted that experiential avoidance would be indirectly associated with IPV perpetration and victimization via all three social problem solving dimensions, was partially supported. Experiential avoidance was associated with the NPO, ICS, and AS social problem solving dimensions. Only NPO and ICS, however, were predictive of IPV perpetration and victimization. Surprisingly, NPO was inversely related to IPV perpetration and victimization. Also notable, experiential avoidance remained directly associated with IPV perpetration and victimization even after including the influences of dysfunction social problem solving.

The finding that NPO was inversely related to IPV perpetration and victimization, such that lower NPO scores were predictive of higher IPV perpetration and victimization, was puzzling. This finding is somewhat inconsistent with results from a previous study demonstrating a positive association between NPO and anger. Notably, however, this same study did not find a significant relationship between NPO and physical aggression [62]. Individuals who score high on NPO have a tendency to view problems as a threat to well-being, doubt their own ability to solve their problems effectively, and exhibit low tolerance for problems [48]. It seems possible that individuals who have a tendency to feel threatened by problems and doubt their own abilities to solve problems effectively are more likely to avoid interpersonal conflict, reducing risk of IPV perpetration and victimization. Conversely, those who view problems as a challenge and are confident in their abilities to handle problems effectively might be more willing to engage in interpersonal conflict, which could increase risk for IPV perpetration and victimization. Given that this is the first study to examine the impact of maladaptive social problem solving on IPV perpetration and victimization, the unexpected inverse relationship between NPO and IPV should be interpreted cautiously. Additional research is needed to further investigate the impact of NPO on IPV perpetration and victimization.

Although experiential avoidance was positively associated with the AS social problem solving dimension, AS was not predictive of IPV perpetration or victimization. Individuals who score high on the AS social problem solving dimension have a tendency to avoid confronting problems, procrastinate, wait passively for problems to resolve, or shift responsibility of problem solving to others [48]. The AS social problem solving dimension has previously been found to be positively associated with anger but not significantly related to physical aggression [62]. In another study, schema avoidance (characterized by social inhibition) did not explain a significant amount of variance in adult interpersonal conflict [26]. Taken together, results from the current and previous studies suggest that experiential avoidance might be associated with a tendency to avoid solving problems, but this avoidance of problem solving does not significantly impact risk of IPV perpetration and victimization. These findings are considered preliminary and additional research is needed to further clarify the relationships between experiential avoidance, the AS social problem solving dimension, and IPV.

The ICS social problem solving dimension, characterized by a tendency to solve problems quickly, carelessly, unsystematically, and impulsively, was positively related to IPV perpetration and victimization. Results suggest that individuals who score high on the ICS social problem solving dimension may be more likely to take a quick, reactive approach to addressing interpersonal problems, increasing IPV risk. This finding is consistent with previous research indicating that heightened impulsivity levels are associated with increased risk for IPV perpetration and victimization among women [80,81]. The current study’s finding is also consistent with results from a previous study that found an association between the ICS social problem solving dimension and physical aggression [62]. Results from the current and previous studies highlight the role of impulsivity, including impulsive or careless problem solving, on risk for IPV perpetration and victimization among women.

Although experiential avoidance indirectly affected IPV perpetration and victimization via the NPO and ICS social problem solving dimensions, experiential avoidance also continued to have a direct effect on IPV perpetration and victimization. Results highlight the important direct and indirect role of emotional processes, such as experiential avoidance, on IPV risk. Additional research is needed to identify other potential mechanisms, beyond maladaptive social problem solving, that account for the relationship between experiential avoidance and IPV perpetration and victimization.

The final hypothesis, that CEA would be indirectly predictive of IPV perpetration and victimization via experiential avoidance and maladaptive social problem solving after controlling for the effects of CPA and CSA, was supported. CEA was the only form of childhood maltreatment that was indirectly associated with IPV perpetration and victimization via experiential avoidance and maladaptive social problem solving. Interestingly, CEA did not have a direct effect on IPV perpetration and victimization after including the impact of experiential avoidance and maladaptive social problem solving. Taken together, these results suggest that affective and cognitive processes affected by CEA can jointly impact risk for IPV perpetration and victimization among women.

Several measurement limitations in the current study should be noted. The SPSI-R:S is a self-report measure, not a performance test, of social problem solving ability. Thus, SPSI-R:S scores are considered to be an estimate of social problem solving ability and may not accurately reflect actual social problem solving ability [35]. The measure also does not define the term “problem” for participants, leaving interpretation of the term up to the individual [35]. Relatedly, the current study did not specifically assess interpersonal social problem solving, as has been attempted in a previous study [67]. It is possible that participants thought about their abilities to solve non-interpersonal problems when responding to the SPSI-R:S, which may be less relevant to IPV risk than ability to solve interpersonal problems. Experiential avoidance was also self-reported by participants who may not be fully aware of how they relate to internal experiences. Assessment of childhood maltreatment was retrospective and subject to memory bias. Further, the CTQ is a brief measure of childhood maltreatment and does not assess the characteristics of the abuse, which could have an impact on the other variables included in the path analytic models [68]. Additional research is needed to investigate how various aspects of CEA, such as ignoring, terrorizing, and exploitation, might differentially impact affective and cognitive processes that could increase IPV risk [26]. Future research should also determine if there are “dosage effects” of CEA and other forms of childhood maltreatment on short and long-term negative outcomes, such that greater exposure and severity of CEA might result in more significant adverse effects [5]. Lastly, due to the limited scope of the project and sensitivity to avoid overburdening participants with too many study demands, the current study did not gather detailed information on participants’ child welfare history and parental involvement. As such, it is not known how participants’ histories of child welfare involvement could further impact their emotional and cognitive processing and how cognitive and emotional impairments could impair participants’ current parenting practices. Future research is needed to address these questions.

There were also some limitations noted with the study’s methodology. The current study was cross-sectional. Thus, the direction or temporal ordering of the relationships between variables could be different than what was predicted. Although the path analytic models tested in the current study were derived from existing theoretical and empirical literature on the relationships between childhood maltreatment, experiential avoidance, maladaptive social problem solving, and IPV, it is possible that IPV experiences could impact experiential avoidance and maladaptive social problem solving or that maladaptive social problem solving could affect experiential avoidance [47]. Future prospective research is needed to examine the temporal relationships between the current study’s variables. Additionally, the study did not assess the impact of witnessing family-of-origin violence, which can affect IPV risk [82]. Protective factors that dampen the impact of childhood maltreatment on short and long-term adverse outcomes, including IPV, also need to be explored [3]. Due to sample size restrictions and power limitations, separate path analyses were conducted for IPV perpetration and victimization. Given the comorbidity between IPV perpetration and victimization, the unique predictors of IPV perpetration and victimization are difficult to ascertain without including both variables in the same model [15]. Relatedly, although our study included over 200 participants, our participant/parameter ratio was slightly below the recommended 10:1 participant/parameter ratio [83]. Our analyses, therefore, may have been slightly underpowered and results should be considered preliminary. Replication of these models with larger sample sizes is needed to determine if similar significant direct and indirect pathways are obtained. Lastly, the study did not gather detailed information on the gender roles and orientations of participants and their partners. It is possible that IPV experienced in non-heterosexual relationships may appear topographically different and/or function differently in these relationships. Furthermore, factors contributing to IPV risk may be different between monogamous heterosexual relationships and other types of relationships. To date, limited research on the IPV characteristics and risk factors in gay, lesbian, bisexual, polyamorous, and transgender relationships has been conducted and future research in this area is desperately needed.

Results from the current study may have important clinical implications. If CEA impacts affective and cognitive processes that can lead to increased IPV risk, then interventions targeting these processes may help to improve interpersonal functioning and reduce IPV risk. For example, mindfulness and acceptance-based interventions that facilitate a non-judgmental, accepting approach toward internal experiences might be useful in addressing experiential avoidance following CEA that could lead to maladaptive interpersonal functioning, including IPV [44]. Additionally, problem solving interventions that promote purposeful, rational, intentional, and adaptive approaches to solving problems might reduce IPV risk [62]. Child-based interventions targeted at addressing emotional and social competency among high-risk children, such as those who have been exposed to CEA and other forms of childhood maltreatment, may also be beneficial in buffering the effects of childhood maltreatment on long-term functioning [15]. Research is needed to develop and evaluate the effectiveness of child and adult-based interventions that target maladaptive affective and cognitive processes that might arise following exposure to childhood maltreatment, especially CEA.

## 5. Conclusions

Despite limitations, the current study was the first to investigate the joint influences of experiential avoidance and maladaptive social problem solving on the link between CEA and IPV after controlling for the effects of other forms of childhood maltreatment. Results indicate that CEA has a unique, indirect impact on IPV perpetration and victimization via experiential avoidance and impulsive/carelessness and negative problem orientation social problem solving. Findings also suggest that both CPA and experiential avoidance directly impact IPV perpetration and victimization even after accounting for the influence of maladaptive social problem solving. Researchers should continue to examine how childhood maltreatment, especially CEA, impairs emotional and cognitive development and leads to long-term adverse outcomes.
